# Inverse association between triglyceride–glucose index and maximal oxygen uptake in US young and middle-aged population: a cross-sectional study

**DOI:** 10.3389/fcvm.2025.1583614

**Published:** 2025-04-09

**Authors:** Bin Zhang, Junxing Lai, Dan Li, Yongfeng Li, Peng Wang, Shangan Cai, Qiang Ren, Dong Li

**Affiliations:** ^1^ Department of Hypertension and Vascular Disease, The First Affiliated Hospital, Sun Yat-sen University, Guangzhou, China; ^2^ Department of Cardiovascular Disease and Clinical Experimental Center, Jiangmen Central Hospital, Jiangmen, China; ^3^ Department of Electrocardiogram, Jiangmen Central Hospital, Jiangmen, China; ^4^ Department of Medical Records, Jiangmen Central Hospital, Jiangmen, China; ^5^ Department of Information, Jiangmen Central Hospital, Jiangmen, China; ^6^ Department of Urology, Jiangmen Central Hospital, Jiangmen, China; ^7^ Department of Intensive Care Unit, Jiangmen Central Hospital, Jiangmen, China

**Keywords:** TyG index, VO_2_max, cross-sectional study, NHANES, adverse

## Abstract

**Background:**

The triglyceride–glucose (TyG) index has been linked to impaired cardiovascular fitness (CVF). However, the available evidence regarding the direct relationship between the TyG index and maximal oxygen uptake (VO_2_max) is limited. This study aims to investigate the association between the TyG index and VO_2_max.

**Methods:**

We conducted a retrospective cross-sectional study involving 3,571 participants who completed a CVF examination as part of the National Health and Nutrition Examination Survey (NHANES) 1999–2004. Data on triglycerides, glucose, and VO_2_max were collected from all participants. The TyG index was calculated using the formula: Ln[triglyceride (TG)(mg/dl) × fasting plasma glucose (FPG)(mg/dl)/2]. Linear regression analysis was utilized to substantiate the research objectives.

**Results:**

The complex sampling design and mobile examination center sample weights were considered. In multivariable linear regression analyses, each 1 unit increase in the TyG index was associated with a decrease in VO_2_max [*β* = −1.24, 95% CI (−1.97, −0.51), *p* = 0.002] when expressed as a continuous variable, independent of confounders. The TyG index was converted into a categorical variable based on four quartiles. Compared with the lowest TyG quintile (Q1: 6.750–7.887), the fully adjusted *β* for Q4 (8.672–12.481) was −1.91 (95% CI: −3.24, −0.57, *p* < 0.007). A significant interaction (*p* = 0.007) between sex and the TyG index for VO_2_max was found in the population using subgroup analysis. The results of the sensitivity analysis remained stable. Mediation analysis showed the direct effect of the TyG index was −1.467 (−2.019, −0.948), with a total effect of −1.813 (−2.377, −1.286). The mediation effect of diastolic blood pressure (DBP), white blood cell count (WBC), and C-reactive protein (CRP) was −0.389 (−0.526, −0.268), −0.308 (−0.432, −0.177), and −0.252 (−0.453, −0.135), respectively. HGB was found to exert a suppressing effect on the relationship between the TyG index and VO_2_max, with a value of 1.469 (1.252, 1.702). The *p*-values for all the above effects were <0.05.

**Conclusions:**

In the US young and middle-aged population, the TyG index was significantly adversely associated with VO_2_max levels. Females may exert an interaction on TyG. Evidence supported DBP, WBC, and CRP as intervening variables through which the TyG index exerts its influence on VO_2_max. HGB may overrule the potential inverse association between the TyG index and VO_2_max.

**NCHS IRB/ERB Protocol Number**: Protocol #98-12.

## Introduction

Cardiovascular diseases (CVD) are a major cause of death and disability worldwide (1–6). With the intensification of population aging, the burden of cardiovascular disease is also constantly increasing (7–10). According to a report by the American Heart Association (AHA), the number of deaths due to cardiovascular diseases exceeds the combined total number of deaths due to cancer and lower respiratory diseases. The crude prevalence of CVD in 2020 increased by 29.01% compared with 2010 (11). Maximal oxygen uptake (VO_2_max), also known as aerobic capacity, is the maximum amount of oxygen an individual can utilize during intense exercise and is a definitive indicator of cardiorespiratory fitness. A decline in VO_2_max is associated with a higher risk of cardiovascular disease and all-cause mortality (12–18). The AHA reports that cardiorespiratory fitness is a better predictor of mortality than many other risk factors, such as smoking, hypertension, or hyperlipidemia. VO_2_max is influenced by a complex network of cardiorespiratory and metabolic systems, and maintaining good VO_2_max is critical for preventing cardiovascular diseases and sustaining overall health.

The triglyceride–glucose (TyG) index, a simple and cost-effective biomarker, has gained increasing attention in recent years for its potential role in assessing insulin resistance (IR) and metabolic disorders. Derived from the product of fasting triglycerides and glucose levels, the TyG index provides valuable insights into the underlying metabolic state of an individual, making it a useful tool in clinical settings. Numerous studies have demonstrated that elevated TyG values are closely associated with an increased risk of CVD, such as hypertension, coronary artery disease, and stroke (19–22). The simplicity of its calculation and strong correlation with IR and CVD make the TyG index a promising marker for the early detection and management of metabolic syndrome.

Despite the extensive research on the TyG and VO_2_max individually, the relationship between the two remains unclear. Therefore, the ultimate goal of this study is to explore the association between the TyG index and VO_2_max.

## Methods

### Study design and population

The data utilized and analyzed in this study were obtained from the National Health and Nutrition Examination Survey (NHANES) database, covering three cycles from 1999 to 2004. NHANES is a continuous survey that employs a multistage, stratified probability sampling design to select a representative sample of the US population, aiming to assess the health and nutritional status of non-institutionalized Americans. The program has been approved by the National Center for Health Statistics (NCHS) Ethics Review Board. Data collection is supervised by the NCHS and adheres to strict ethical standards with informed consent obtained from all participants. The NHANES data set includes various health indicators such as demographic characteristics, physical examination results, laboratory findings, and dietary habits. The exclusion criteria for this study included (1) incomplete treadmill aerobic exercise for VO_2_max testing, (2) missing or unavailable fasting glucose data, (3) missing or unavailable fasting triglyceride data, and (4) fasting glucose weight of zero or missing.

### Variables and covariates

Only participants aged 12–49 years were eligible for the cardiovascular fitness (CVF) examination, making the study population representative of the young and middle-aged population in the United States. During the CVF examination, participants were typically instructed to perform aerobic exercise on a treadmill to measure VO_2_max. NHANES utilized enzymatic assays to measure fasting plasma triglyceride (TG) and fasting plasma glucose (FPG) levels from blood samples analyzed using an automatic analyzer. These samples were collected from individuals who had fasted for at least 8 h but no more than 24 h. The TyG index was calculated using the formula Ln[TG (mg/dl) × FPG (mg/dl)/2] (23).

Various potential covariates were assessed based on clinical practice and literature review (24, 25), including age, sex/gender, race/ethnicity, smoking status, alcohol consumption, hypertension, diabetes, body mass index (BMI), waist circumference, glycated hemoglobin hemoglobin A1c (Ghb), C-reactive protein (CRP), albumin (ALB), creatinine (CR), white blood cell count (WBC), red blood cell count (RBC), hemoglobin (HGB), low-density lipoprotein cholesterol (LDL-C), high-density lipoprotein cholesterol (HDL-C), TG, and total cholesterol (TC). Ethnicity was categorized into non-Hispanic White, non-Hispanic Black, Mexican American, and other races. Smoking status was divided into non-smokers (<100 cigarettes smoked) and smokers (≥100 cigarettes smoked). Alcohol consumption was self-reported and categorized as non-drinkers (<12 drinks in a lifetime) and drinkers (at least 12 drinks in a year or at least 12 drinks in the past year). Hypertension and diabetes were defined based on self-reported diagnosis of the condition.

Age, gender, and ethnicity were acquired from demographic data. BMI, waist circumference, systolic blood pressure (SBP), and diastolic blood pressure (DBP) were acquired from examination data. CRP, HGB, LDL, HDL, TC, Ghb, FPG, and TG were acquired from laboratory data.

### Statistical analysis

Our analysis accounted for the complex sampling design and fasting plasma glucose sample weights. For the combined NHANES 1999–2000 and 2001–2002 datasets, we used the Fasting Subsample 4 Year MEC Weight (WTSAF4YR). For NHANES 2003–2004, we used the Fasting Subsample 2 Year MEC Weight (WTSAF2YR). Sampling weights were calculated as follows: weights were set for the 1999–2002 period at 2/3 × WTSAF4YR and for the 2003–2004 period at 1/3 × WTSAF2YR. Histogram distribution, or Q–Q plot, or the Kolmogorov–Smirnov test was used to determine whether variables were normally distributed. Categorical data are presented as unweighted counts and frequencies (%), while continuous variables are presented as medians and interquartile ranges (IQR) from the 25th to the 75th percentiles. Differences between multiple groups of continuous variables were assessed using the one-way ANOVA test (for normal distribution) or Kruskal–Wallis H test (for skewed distribution) to compare differences among groups. Chi-squared tests were employed to assess significant differences between categorical variables.

A weighted multivariate linear regression analysis was employed to determine the association between the TyG index and VO_2_max, with results expressed as beta (*β*) and 95% confidence intervals (CIs). We constructed four models: the unadjusted model; Model 1 adjusted for demographic variables, including age, gender, and ethnicity; Model 2 adjusted for smoking status, alcohol consumption, hypertension, diabetes, BMI, and waist circumference; and Model 3 adjusted for Ghb, CRP, ALB, CR, WBC, RBC, and HGB. In addition to using the TyG index as a continuous variable, we also converted TyG into a categorical variable according to the four quartiles and calculated the *p*-value for trend to verify the results of the TyG index as a continuous variable and to examine the possibility of non-linearity.

The weighted restricted cubic splines (RCS) with unadjusted and adjusted covariates in Model 3 were employed to further explore the linearity relationship between the TyG index and VO_2_max. Subgroup and sensitivity analyses were conducted to ensure the robustness of our results. We performed interaction and subgroup analyses using linear regression models stratified by gender, ethnicity, smoking status, alcohol consumption, and obesity. The non-Hispanic White population was utilized for the sensitivity study. Linear regression models and RCS were also performed in sensitivity analyses. Mediation analysis was conducted to investigate whether certain variables acted as mediators in the association between the TyG index and VO_2_max.

All statistical analyses in this study were performed using R Statistical Software (Version 4.4.1, http://www.R-project.org) and the Free Statistics analysis platform (Version 1.9.2; Beijing, China; http://www.clinicalscientists.cn/freestatistics). Descriptive statistics were computed for all participants, and statistical significance was defined as a two-sided *p*-value of <0.05.

## Results

### Study population

**Figure 1:**
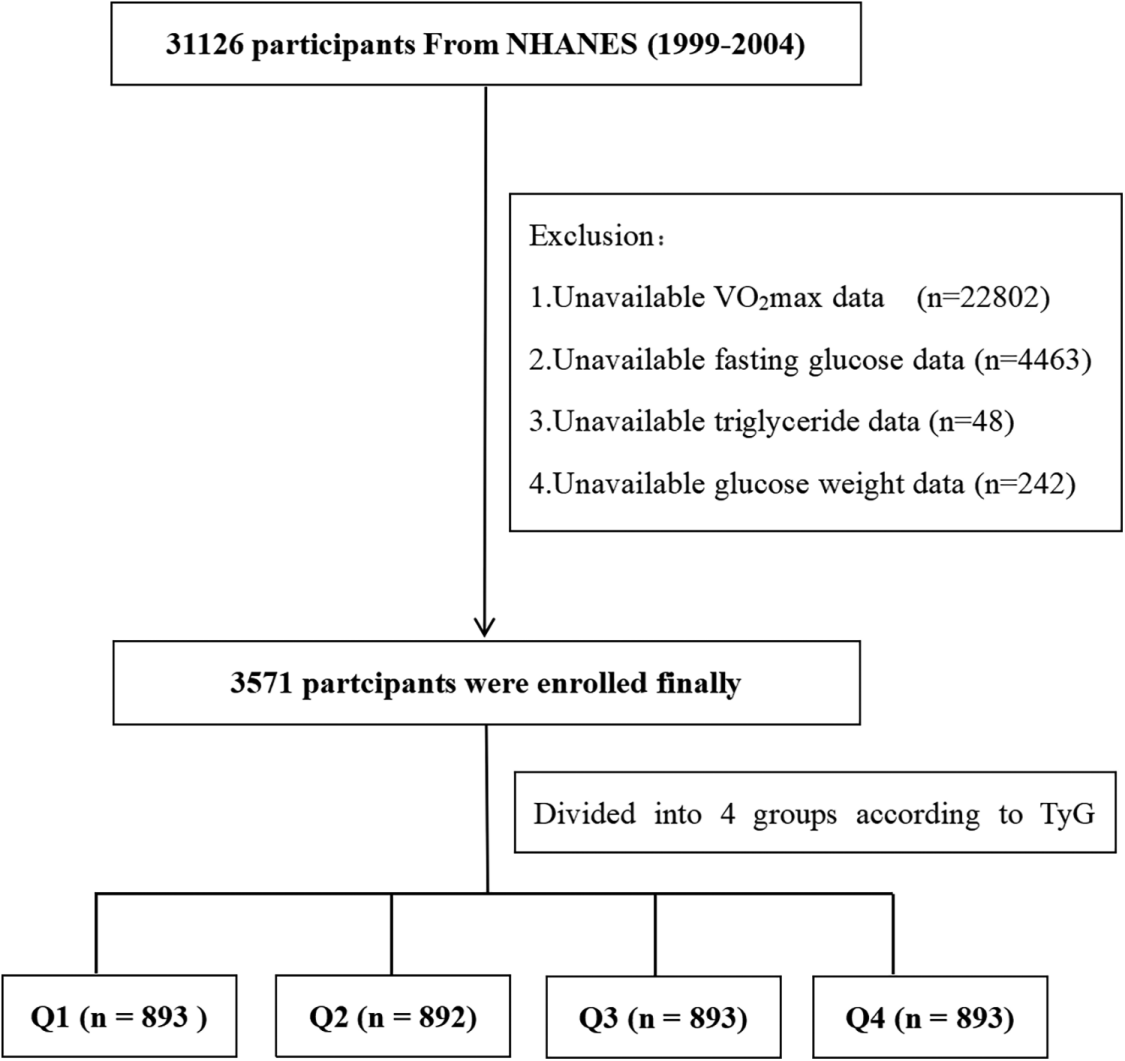
Flow diagram of the screening and enrollment of study participants.

This study utilized data from three NHANES cycles, 1999–2000, 2001–2002, and 2003–2004, with a total of 31,126 participants completing the survey. We excluded participants with missing or unavailable data on VO_2_max (*n* = 22,802), fasting glucose data (*n* = 4,463), and triglyceride data (*n* = 48). In addition, participants without data on fasting glucose sample weights (*n* = 242) were excluded. Consequently, the final sample consisted of 3,571 participants (Figure 1). These participants represent the population aged 12–49 years old in the United States. The study population had a median age of 18 (IQR: 15–30) years, with 1,900 (53.21%) male participants and 1,230 (34.44%) non-Hispanic White participants (Table 1).

**Table 1:** Baseline characteristics of the study population according to the quartiles of the TyG index.

Characteristics	Total (*n* = 3,571)	Q1 (*n* = 893)	Q2 (*n* = 892)	Q3 (*n* = 893)	Q4 (*n* = 893)
Gender (%)					
Male	1,900 (53.21)	430 (48.15)	429 (48.09)	471 (52.74)	570 (63.83)
Female	1,671 (46.79)	463 (51.85)	463 (51.91)	422 (47.26)	323 (36.17)
Ethnicity (%)					
Non-Hispanic White	1,230 (34.44)	237 (26.54)	279 (31.28)	336 (37.63)	378 (42.33)
Non-Hispanic Black	939 (26.30)	388 (43.45)	256 (28.7)	181 (20.27)	114 (12.77)
Mexican American	1,111 (31.11)	208 (23.29)	267 (29.93)	312 (34.94)	324 (36.28)
Other	291 (8.15)	60 (6.72)	90 (10.09)	64 (7.17)	77 (8.62)
Hypertension (%)					
No	3,368 (94.32)	871 (97.54)	862 (96.64)	852 (95.41)	783 (87.68)
Yes	203 (5.68)	22 (2.46)	30 (3.36)	41 (4.59)	110 (12.32)
Diabetes (%)					
No	3,524 (98.68)	893 (100)	887 (99.44)	891 (99.78)	853 (95.52)
Yes	47 (1.32)	0 (0)	5 (0.56)	2 (0.22)	40 (4.48)
CKD (%)					
No	3,554 (99.52)	892 (99.89)	890 (99.78)	887 (99.33)	885 (99.1)
Yes	17 (0.48)	1 (0.11)	2 (0.22)	6 (0.67)	8 (0.9)
Cancer, *n* (%)					
No	3,546 (99.30)	889 (99.55)	885 (99.22)	884 (98.99)	888 (99.44)
Yes	25 (0.70)	4 (0.45)	7 (0.78)	9 (1.01)	5 (0.56)
Alcohol, *n* (%)					
No	2,314 (64.80)	706 (79.06)	644 (72.2)	556 (62.26)	408 (45.69)
Yes	1,257 (35.20)	187 (20.94)	248 (27.8)	337 (37.74)	485 (54.31)
Smoking status (%)					
No	2,976 (83.34)	815 (91.27)	798 (89.46)	720 (80.63)	643 (72)
Yes	595 (16.66)	78 (8.73)	94 (10.54)	173 (19.37)	250 (28)
Age (years)	18.00 (15.00, 30.00)	17.00 (14.00, 20.00)	17.00 (14.00, 25.00)	19.00 (15.00, 31.00)	26.00 (17.00, 37.00)
Weight (kg)	68.90 (57.20, 82.90)	63.00 (53.10, 72.90)	64.00 (54.48, 75.12)	70.10 (58.00, 83.90)	81.20 (68.40, 94.50)
Height (cm)	167.60 (160.70, 175.00)	166.40 (159.90, 174.10)	165.95 (159.98, 173.20)	167.80 (160.80, 175.50)	169.80 (162.40, 176.90)
BMI (kg/m^2^)	24.03 (20.87, 28.42)	21.96 (19.70, 25.02)	22.66 (20.18, 26.15)	24.30 (21.32, 28.95)	27.94 (24.31, 31.94)
Waist (cm)	82.90 (74.00, 95.20)	76.30 (70.30, 84.80)	78.90 (72.10, 88.10)	84.20 (75.20, 96.00)	95.50 (85.40, 104.50)
SBP (mmHg)	111.00 (104.00, 118.00)	108.00 (102.00, 115.00)	109.00 (103.00, 116.00)	112.00 (105.00, 119.00)	115.00 (108.00, 123.00)
DBP (mmHg)	66.00 (59.00, 73.00)	63.00 (56.00, 70.00)	65.00 (58.00, 71.25)	67.00 (59.00, 74.00)	70.00 (62.00, 77.00)
Ghb (%)	5.20 (5.00, 5.40)	5.10 (5.00, 5.30)	5.20 (5.00, 5.30)	5.20 (5.00, 5.30)	5.20 (5.10, 5.50)
C-peptide (pmol/ml)	0.63 (0.48, 0.83)	0.52 (0.41, 0.66)	0.58 (0.46, 0.73)	0.65 (0.53, 0.84)	0.81 (0.65, 1.07)
Insulin (uU/ml)	9.60 (6.69, 14.23)	7.88 (5.47, 11.04)	8.72 (6.37, 12.33)	9.79 (7.03, 14.58)	12.90 (8.92, 19.83)
CRP (mg/dl)	0.08 (0.03, 0.24)	0.05 (0.02, 0.14)	0.05 (0.02, 0.18)	0.08 (0.03, 0.27)	0.17 (0.06, 0.38)
ALB (ug/ml)	8.50 (4.80, 16.95)	9.00 (5.30, 17.80)	8.50 (4.90, 17.83)	8.20 (4.60, 15.70)	8.00 (4.60, 16.80)
CR (mg/dl)	154.00 (103.00, 215.00)	163.00 (108.00, 229.00)	153.00 (100.00, 217.00)	150.00 (100.00, 209.00)	152.00 (104.00, 207.00)
WBC (10^−9^/L)	6.10 (5.10, 7.40)	5.60 (4.60, 6.80)	6.00 (5.00, 7.20)	6.30 (5.30, 7.60)	6.60 (5.60, 7.90)
RBC (10^−12^/L)	4.85 (4.49, 5.19)	4.69 (4.37, 5.05)	4.80 (4.43, 5.13)	4.87 (4.53, 5.19)	5.03 (4.66, 5.35)
HGB (g/dl)	14.40 (13.50, 15.50)	13.90 (13.00, 15.00)	14.20 (13.40, 15.20)	14.50 (13.60, 15.50)	15.00 (14.00, 16.00)
Glucose (mg/dl)	91.20 (86.50, 96.80)	87.80 (83.50, 92.50)	90.50 (85.70, 95.43)	92.40 (87.70, 97.30)	95.40 (89.80, 101.90)
TC (mg/dl)	168.00 (147.00, 194.00)	150.00 (135.00, 169.00)	162.00 (144.00, 181.00)	172.00 (153.00, 194.00)	196.00 (170.00, 224.00)
HDL (mg/dl)	4.91 (4.06, 39.00)	4.27 (3.67, 42.00)	4.65 (3.98, 44.00)	5.02 (4.22, 39.00)	5.64 (4.65, 31.00)
Triglyceride (mg/dl)	81.00 (59.00, 119.00)	48.00 (42.00, 54.00)	69.50 (63.00, 76.00)	97.00 (87.00, 108.00)	162.00 (137.00, 207.00)
LDL (mg/dl)	97.00 (80.00, 119.50)	84.00 (70.00, 100.00)	95.00 (78.00, 113.00)	103.00 (85.00, 122.00)	116.00 (94.00, 141.00)
Predicted VO_2_max (ml/kg/min)	40.22 (33.04, 48.73)	43.23 (35.80, 51.61)	41.97 (34.34, 49.86)	39.83 (32.70, 48.12)	36.60 (29.87, 43.55)
VO_2_max (ml/kg/min)	40.51 (35.14, 47.04)	41.81 (36.33, 48.28)	41.20 (35.28, 48.81)	40.03 (34.68, 46.38)	39.40 (34.40, 45.30)
TyG index	8.22 (7.89, 8.63)	7.67 (7.53, 7.78)	8.05 (7.97, 8.13)	8.40 (8.30, 8.51)	8.95 (8.76, 9.21)

Data are presented as unweighted number and percentage for the categorical variables. The continuous variables were presented as median and quartile. CKD, chronic kidney disease; BMI, body mass index; SBP, systolic blood pressure; DBP, diastolic blood pressure; Ghb, glycohemoglobin; CRP, C-reactive protein; ALB, albumin; CR, creatinine; WBC, white blood cell; RBC, red blood cell; HGB, hemoglobin; TC, total cholesterol; HDL, high-density lipoprotein cholesterol; LDL, low-density lipoprotein cholesterol; TyG index, triglyceride–glucose index. *p*-values in bold are <0.05.

### Baseline characteristics according to the quartiles of the TyG index

Table 1 presents the baseline characteristics of participants by quantiles of the TyG index: Q1 (*n* = 893, the lowest quartile), 6.750 ≤ TyG index ≤ 7.887; Q2 (*n* = 892, the second quartile), 7.887 < TyG index ≤ 8.216; Q3 (*n* = 893, the upper quartile), 8.216 < TyG index ≤ 8.672; and Q4 (*n* = 893, the highest quartile), 8.672 < TyG index ≤ 12.481). Compared with participants in the Q1 group, those in the Q4 group were generally elderly, predominantly male, more likely to be Mexican American participants, and had higher BMI, waist circumference, SBP, DBP, CRP, HGB, LDL, HDL, C-peptide, FPG, and TG and lower VO_2_max levels (*p* < 0.01).

### Relationship between the TyG index and VO_2_max

The univariable analysis demonstrated that age, sex, ethnicity, alcohol consumption, hypertension, diabetes, Ghb, CRP, ALB, CR, WBC, RBC, HGB, and TyG were all associated with VO_2_max (results are shown in Supplementary Table S1).

In weighted multivariable linear regression analyses examining the association between TyG index and VO_2_max, with adjustment for potential confounders (Table 2, Model 3), the TyG index expressed as a continuous variable (increasing per 1 unit) was associated with decreased VO_2_max [*β* = −1.24, 95% CI (−1.97, −0.51), *p* = 0.002]. An inverse relationship between the TyG index with VO_2_max was also observed after adjusting for potential confounders when the TyG index was expressed as a four quartile-categorized variable. Compared to Q1, the adjusted *β*s in Q2, Q3, and Q4 were −0.50 (95% CI:−1.91, 0.90, *p* = 0.465), −1.59 (95% CI: −2.62, −0.56, *p* = 0.004), and −1.91 (95% CI: −3.24, −0.57, *p* < 0.007), respectively (Table 2, Model 3).

**Table 2:** Association between the TyG index and VO_2_max (weighted).

TyG index	Unadjusted		Model 1		Model 2		Model 3	
	*β* (95% CI)	*p*-value	β (95% CI)	*p*-value	β (95% CI)	*p*-value	β (95% CI)	*p*-value
Continuous	−1.04 (−1.68, −0.39)	0.002	−1.98 (−2.68, −1.27)	<0.001	−1.20 (−1.92, −0.47)	0.002	−1.2 4 (−1.97, −0.51)	0.002
Quartile								
Q1 (6.750–7.887)	1 (reference)		1 (reference)		1 (reference)		1 (reference)	
Q2 (7.887–8.216)	−0.96 (−2.47, 0.56)	0.21	−0.6 (−1.95, 0.75)	0.377	−0.47 (−1.80, 0.86)	0.475	−0.50 (−1.91, 0.90)	0.465
Q3 (8.216–8.627)	−1.85 (−3.02, −0.67)	0.003	−2.23 (−3.21, −1.25)	<0.001	−1.64 (−2.65, −0.63)	0.002	−1.59 (−2.62, −0.56)	0.004
Q4 (8.627–12.481)	−1.98 (−3.22, −0.73)	0.003	−3.09 (−4.37, −1.81)	<0.001	−1.91 (−3.21, −0.62)	0.005	−1.91 (−3.24, −0.57)	0.007
*P* for trend		0.001		<0.001		0.001		0.001

Model 1: adjusted for age, gender, and ethnicity.

Model 2: adjusted for Model 1 + smoking status, alcohol, hypertension, diabetes, BMI, and waist circumference.

Model 3: adjusted for Model 2 + Ghb, CRP, ALB, CR, WBC, RBC, and HGB.

**Figure 2:**
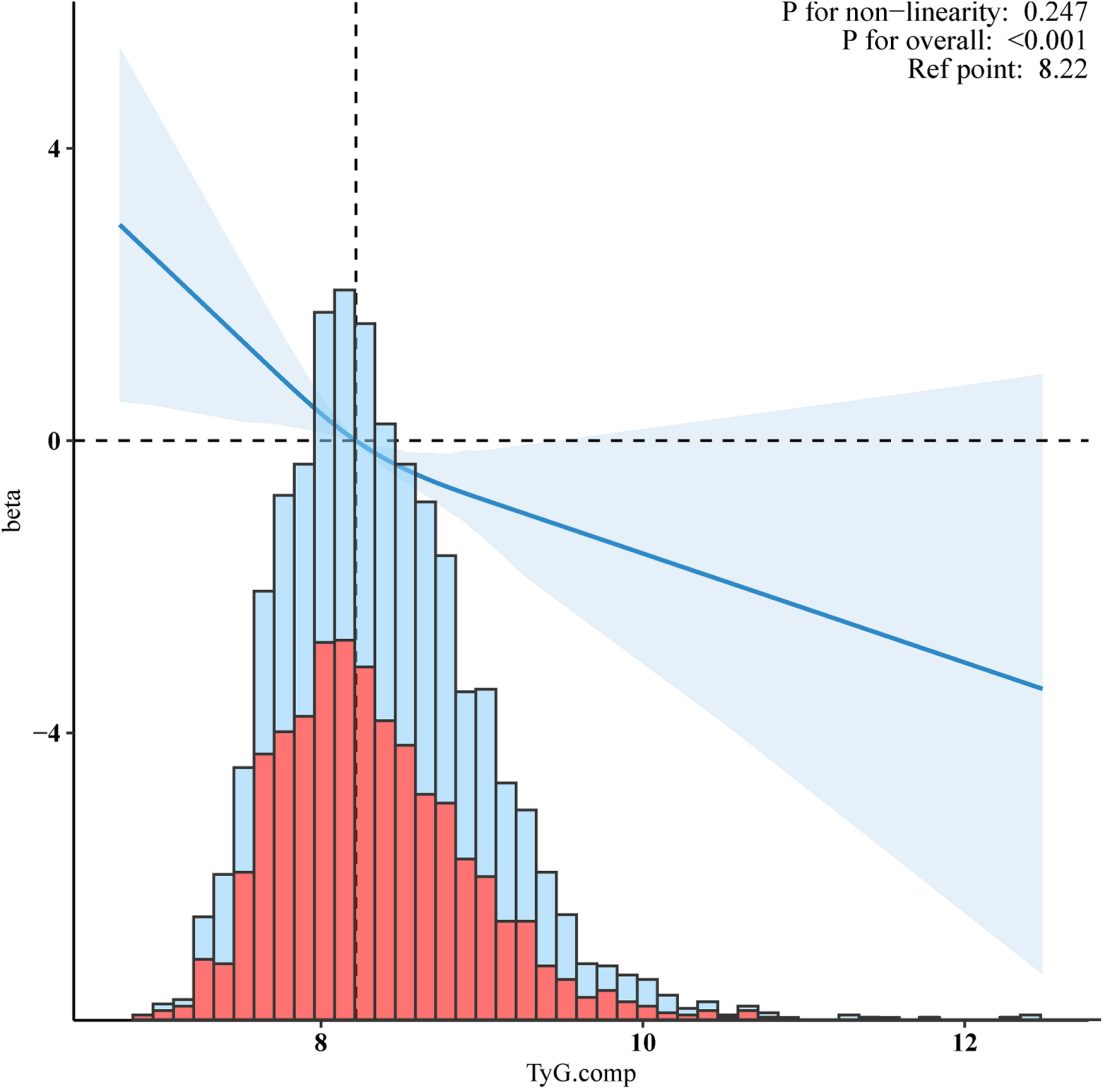
Negative linear relationship between the TyG index and VO_2_max. Adjusted for age, gender, ethnicity, smoking status, alcohol, hypertension, diabetes, BMI, waist circumference, Ghb, CRP, ALB, CR, WBC, RBC, and HGB.

Restricted cubic splines (RCS) with adjusted multivariable suggest a straightforward negative linear relationship between the TyG index and VO_2_max (Figure 2, *p* for non-linearity = 0.247.) As the level of the TyG index increases, the value of VO_2_max shows a downward trend. The solid lines indicate the multivariate-adjusted hazard ratios, and the dashed lines indicate the 95% CIs derived from restricted cubic spline regression. The linear regression was adjusted for age, gender, ethnicity, smoking status, alcohol consumption, hypertension, diabetes, BMI, waist circumference, Ghb, CRP, ALB, CR, WBC, RBC, and HGB.

### Subgroup analysis

**Figure 3:**
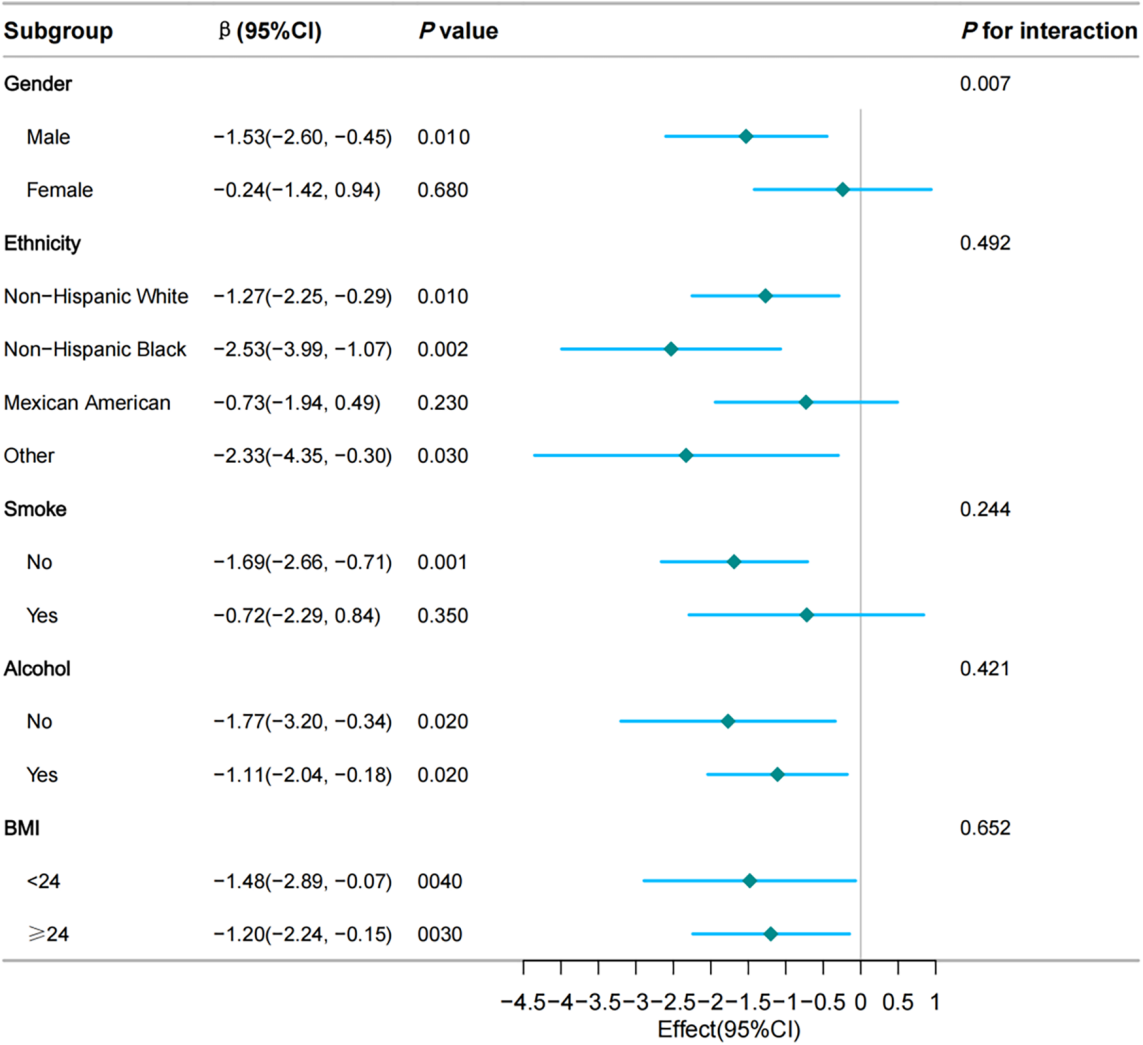
Subgroup and interaction analyses of the TyG index and VO_2_max. Multivariable weighted logistic model adjusted for hypertension, diabetes, waist circumference, Ghb, CRP, ALB, CR, WBC, RBC, and HGB.

To further investigate the association between the TyG index and VO_2_max across a diverse population, we stratified the population based on gender, ethnicity, smoking status, alcohol consumption, and obesity. Due to a limited number of cases of CVD, stroke, chronic kidney disease (CKD), and cancer, these factors were not considered as subgroup criteria. Similarly, since only participants aged 12–49 years were eligible for the CVF examination, making the study population representative of young and middle-aged adults in the United States, age was also not considered as a subgroup criterion. Weighted multivariable linear regression analysis presented no significant interactions between ethnicity, smoking status, alcohol consumption, obesity, and the TyG index for VO_2_max (all *p* for interaction ≥ 0.05, Figure 3). while a significant interaction (*p* = 0.007, Figure 3) between gender and the TyG index for VO_2_max was noted.

### Sensitivity analysis

**Figure 4:**
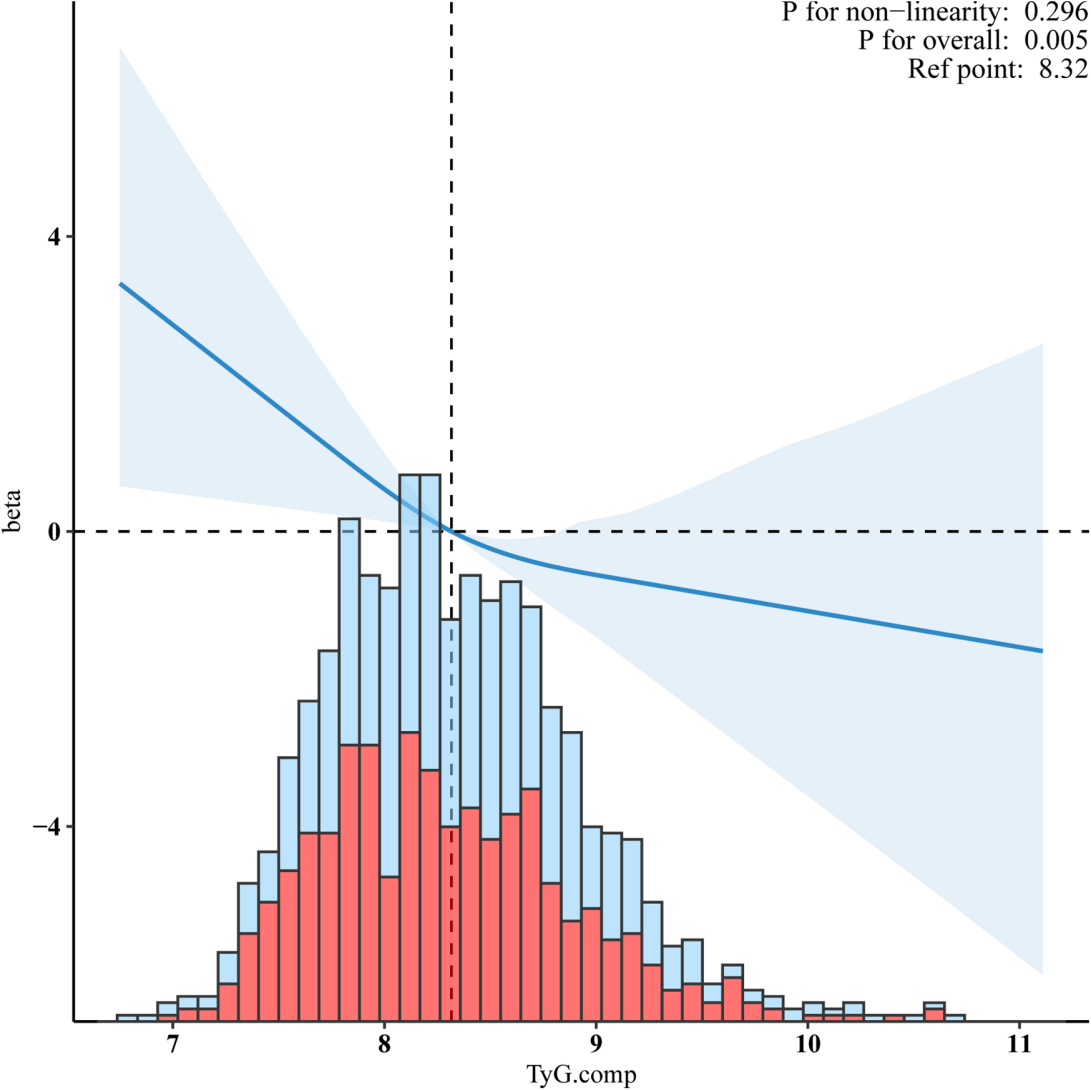
Sensitivity analyses for a negative linear relationship between the TyG index and VO_2_max in the non-Hispanic white population. Adjusted for age, gender, ethnicity, smoking status, alcohol, hypertension, diabetes, BMI, waist circumference, Ghb, CRP, ALB, CR, WBC, RBC, and HGB.

The basic characteristics of the non-Hispanic White population and the results of sensitivity analyses are presented in Table 3. Compared to Q1, the adjusted *β* in Q4 was −0.77 (95% CI:−1.24, −0.3, *p* = 0.001). The TyG index, expressed as a continuous variable, was also inversely associated with VO_2_max (*β* = −1.44, 95%CI:−2.39, −0.4, *p* = 0.003). Figure 4 shows the RCS indicating the negative linear relationship between the TyG index and VO_2_max (*p* for non-linearity = 0.247).

**Table 3:** Sensitivity analysis of the association between the TyG index and VO_2_max (unweighted).

TyG index	Unadjusted		Model 1		Model 2		Model 3	
	β (95% CI)	*p*-value	β (95% CI)	*p*-value	β (95% CI)	*p*-value	β (95% CI)	*p*-value
Continuous	−1.5 (−2.43, −0.57)	0.002	−2.44 (−3.33, −1.55)	<0.001	−1.56 (−2.5, −0.63)	0.001	−1.44 (−2.39, −0.48)	0.003
Quartile								
Q1 (≤8.16)	1 (reference)		1 (reference)		1 (reference)		1 (reference)	
Q2 (8.16–8.58)	−1.8 (−3.41, −0.19)	0.029	−0.95 (−2.4, 0.5)	0.197	−0.88 (−2.31, 0.55)	0.23	−0.88 (−2.32, 0.55)	0.228
Q3 (8.58–9.04)	−2.83 (−4.37, −1.28)	<0.001	−2.71 (−4.11, −1.32)	<0.001	−2.16 (−3.55, −0.77)	0.002	−2.04 (−3.44, −0.63)	0.005
Q4 (≥9.04)	−2.91 (−4.43, −1.4)	<0.001	−3.54 (−4.95, −2.14)	<0.001	−2.41 (−3.85, −0.97)	0.001	−2.21 (−3.68, −0.73)	0.003
*P* for trend	−0.92 (−1.4, −0.45)	<0.001	−1.24 (−1.68, −0.79)	<0.001	−0.84 (−1.3, −0.38)	<0.001	−0.77 (−1.24, −0.3)	0.001

Model 1: adjusted for age, gender, and ethnicity.

Model 2: adjusted for Model 1 + smoking status, alcohol, hypertension, diabetes, CKD, BMI, and waist circumference.

Model 3: adjusted for Model 2 + HDL, LDL, Ghb, CRP, ALB, CR, WBC, RBC, and HGB.

### Mediation analysis

**Figure 5:**
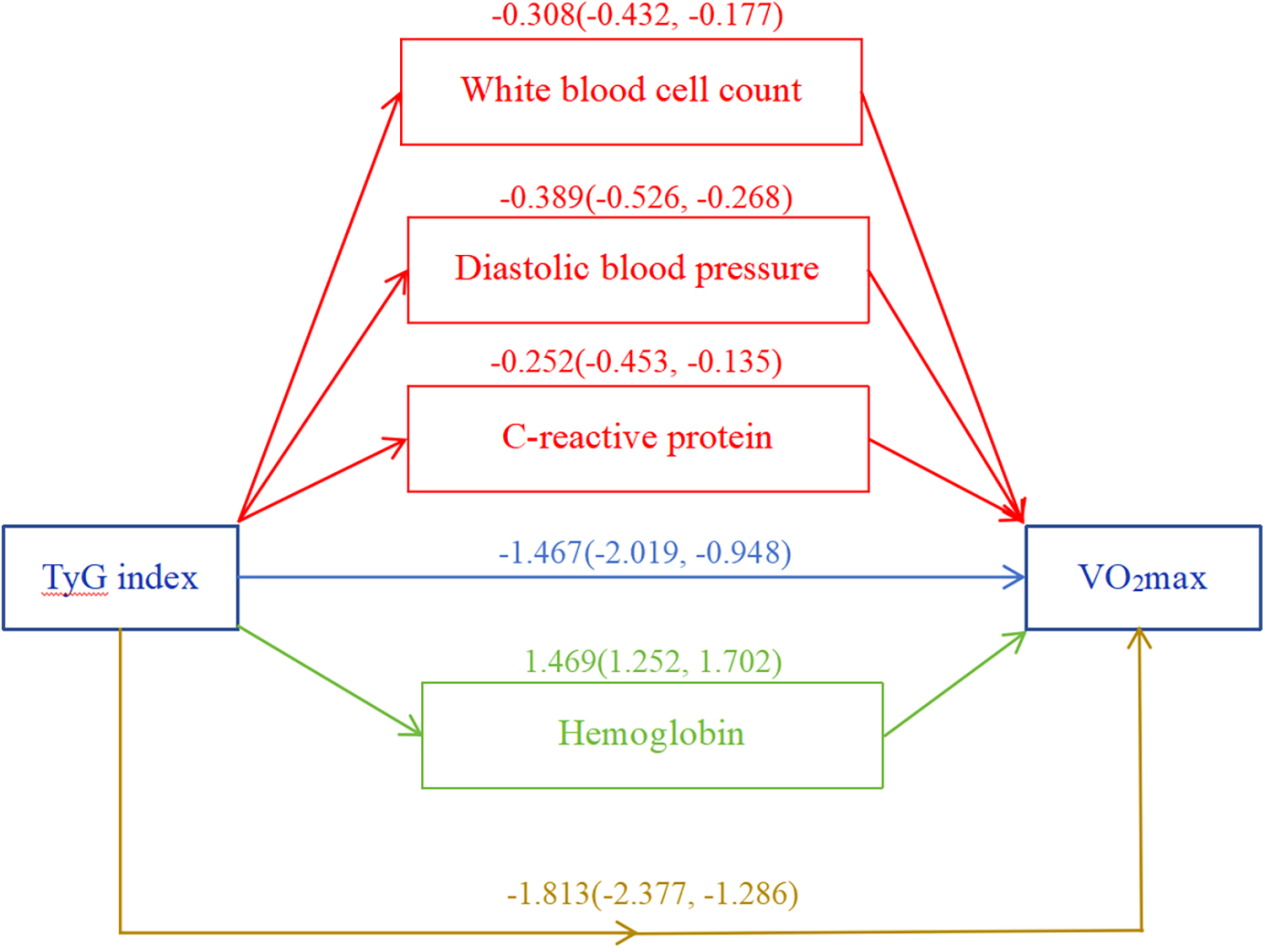
Mediation analysis. White blood cell count, diastolic blood pressure, C-reactive protein, and hemoglobin were included as covariate variables. *p < 0.05.

DBP, WBC, CRP, and HGB were identified as mediating variables to explore their influence on the association between the TyG index and VO_2_max. The direct effect of the TyG index was −1.467 (−2.019, −0.948), with a total effect of −1.813 (−2.377, −1.286). The mediation effect of DBP, WBC, and CRP was −0.389 (−0.526, −0.268), −0.308 (−0.432, −0.177), and −0.252 (−0.453, −0.135), respectively. HGB was found to exert a suppressing effect on the relationship between the TyG index and VO_2_max, with a value of 1.469 (1.252, 1.702). The *p*-values for all the above effects were <0.05 (Figure 5).

## Discussion

In this large population-based US retrospective cross-sectional study, we revealed a negative linear association between the TyG index and VO_2_max in a young and middle-aged population for the first time. Similar patterns of association were observed for subsequent subgroup and sensitivity analyses. Additionally, we found a significant interaction in the relationship between the TyG index and VO_2_max in females. Notably, DBP, WBC, CRP, and HGB were found to mediate the relationship between TyG index and VO_2_max.

In this study, we did not find a statistically significant association between the TyG index and all-cause or cardiovascular mortality. This may be due to our study population only consisting primarily of young and middle-aged individuals (aged 12–49 years), as this is the only group that completed the VO_2_max test, with the majority not having concurrent cardiovascular or other underlying conditions. In addition, it was noteworthy that our study was conducted 20 years ago (1999–2004). Thus, further long-term prospective studies are needed to track the impact of the TyG index on VO_2_max decline and subsequent cardiovascular events in this population.

Previous studies have shown that the TyG index is associated with coronary artery calcification, subclinical atherosclerosis, and arterial stiffness (20, 21, 26, 27), indicating that the TyG index is closely related to the incidence of CVD. A decline in VO_2_max is associated with a higher risk of cardiovascular disease and all-cause mortality, which serves as an important indicator for cardiovascular health stratification (13–17). To our knowledge, previous studies have primarily focused on the relationship between TyG and CVF which was defined through VO_2_max (24), but no studies have directly investigated the correlation between TyG and VO_2_max. Additionally, most studies on the TyG index and CVD have primarily focused on elderly individuals (19, 20), who have a high prevalence of chronic underlying diseases, rather than on young and middle-aged populations who have a lower prevalence of chronic underlying diseases and are generally healthier. Therefore, we chose to directly explore the relationship between TyG and VO_2_max in a young and middle-aged population, rather than CVF. Our current study found a significant adverse linear relationship between TyG and VO_2_max in this population, where individuals with higher TyG index values are more likely to experience a decline in VO_2_max. The results from both subgroup and sensitivity analyses remained robust and further confirmed the relationship.

A meta-analysis of 87 studies indicated that females with metabolic syndrome have a higher risk of CVD than males (28). Additionally, among individuals with impaired glucose tolerance, females have a higher risk of coronary heart disease than males (29). Consequently, females are generally at a higher risk of CVD compared with males. However, in our study, we found a significant negative linear association between the TyG index and impaired CVF in males. The reason for this difference may be that most females in previous studies were elderly and were in late perimenopause or menopause, whereas our study included younger and middle-aged females who still benefit from the protective effects of estrogen. Estrogens play a vital protective role in the cardiovascular system and metabolic health (30–35). They increase HDL-C levels and decrease LDL-C levels, thereby improving the lipid profile and reducing the risk of CVD. Additionally, estrogens maintain the structural and functional integrity of vascular endothelial cells and promote vasodilation by influencing the synthesis of vasodilatory substances such as nitric oxide (NO), thereby maintaining normal vascular function and improving arterial compliance (35). This is crucial for preventing atherosclerosis and cardiovascular disease. Furthermore, estrogens improve insulin sensitivity through modulation of endothelium-dependent and calcium-dependent processes (35, 36). Through multiple mechanisms, estrogens provide broad protection for the cardiovascular system and have positive effects on metabolic health. Given the age range (12–49 years old) of our study population, females in this age group have suffecient estrogen levels to reduce insulin resistance, which may reduce insulin resistance and explain the differences in our results. This may be the reason for the significant interaction between gender and the TyG index for VO_2_max in our study.

The results of the intermediary analysis suggested that part of the harmful effects of the TyG index on VO_2_max were realized through its effects on DBP, WBC, and CRP. Simultaneously, the TyG index enhanced the oxygen transport capacity by increasing HGB, thereby enhancing VO_2_max. There was a lack of dedicated research exploring the correlation between the TyG index and HGB. However, earlier studies suggested that individuals with structural anomalies in HGB typically exhibited lower insulin resistance compared with those with normal HGB levels (37). This finding might have constituted a supporting element for the outcomes of our intermediary analysis. However, further research is necessary to substantiate the potential relationship between the TyG index and HGB.

Previous studies have revealed the role of the TyG index in the occurrence, development, and prognosis of CVD (38–40). To date, no study has examined VO_2_max in relation to the TyG index. In our study population, we confirmed the negative correlation between triglyceride–glucose index and VO_2_max in the context of a population-based cohort on NHANES 1999–2004 even after accounting for confounders. Moreover, these results are generally robust according to subgroups and sensitivity analyses. Although the cross-sectional nature of our study means that conclusions regarding cause–effect relationships require careful interpretation, the results obtained by mediation analysis are consistent with previously reported mechanisms and suggest that the impacts of TyG index on VO_2_max are, to a significant extent, indirectly mediated by DBP, WBC, and CRP. While HGB may overrule the potential inverse association between the TyG index on VO_2_max, future studies are needed to confirm this.

## Limitations

There are several limitations in the present study. An important limitation of this study is the failure to adjust for the impact of dietary factors on VO_2_max. Dietary habits are key factors that affect metabolic health and cardiopulmonary function. Future studies need to incorporate detailed dietary data and use multivariate analysis and mediation analysis to assess the impact of dietary factors on VO_2_max and metabolic health. Moreover, due to the cross-sectional design of our study, the conclusions drawn need to be validated by prospective studies. Although we performed a correlation analysis involving the TyG index with all-cause and cardiovascular mortality, we did not perform a correlation analysis of the TyG index with adverse cardiovascular events because of limitations in the NHANES database. Furthermore, our patients were all from the United States. Studies from other countries or populations are necessary to confirm our findings. Finally, an observational study is not completely the same as a randomized controlled trial (RCT). Therefore, the obtained findings may differ from the expected results of RCT and should be interpreted as being affected in life activities. Notwithstanding these limitations, our data sufficiently investigate how the TyG index is associated with VO_2_max, providing additional evidence on this topic.

## Conclusions

This analysis of NHANES data has demonstrated that in young and middle-aged populations, the TyG index significantly has a negative linear relationship with VO_2_max, independent of confounders. Additionally, sex may exert an influence on the TyG index, with males being more susceptible to declined VO_2_max under similar TyG index conditions. The effect of the TyG index on VO_2_max was partially transmitted through DBP, WBC, and CRP, suggesting that these factors acted as mediators. While HGB may overrule the potential inverse association between TyG index on VO_2_max, future studies are needed to confirm this.

## Data Availability

The original contributions presented in the study are included in the article/Supplementary Material, further inquiries can be directed to the corresponding author.
