# Supplementary material for: Inverse association between triglyceride–glucose index and maximal oxygen uptake in US young and middle-aged population: a cross-sectional study
Source: Front Cardiovasc Med. 2025 Apr 9;12:1583614. doi: 10.3389/fcvm.2025.1583614 (PMC12014587; doi:10.3389/fcvm.2025.1583614)
Supplement: Supplementary file 1 [file Table1.docx]

TableS1 univariable analysis

| **Covariates** | **β(95%CI)** | ***P*-value** |
| --- | --- | --- |
| Gender | -7.43 (-8.03,-6.83) | < 0.001 |
| Age | -0.15 (-0.18,-0.11) | < 0.001 |
| Ethnicity: |  |  |
| ref.=Non-Hispanic White |  |  |
| Non-Hispanic Black | -0.59 (-1.42,0.25) | 0.17 |
| Mexican American | 0.65 (-0.15,1.45) | 0.112 |
| Other | -0.33 (-1.58,0.93) | 0.612 |
| Smoking status | -0.54 (-1.4,0.33) | 0.225 |
| Alcohol | -2.41 (-3.08,-1.74) | < 0.001 |
| HBP | -1.67 (-3.07,-0.27) | 0.019 |
| DM | -4.23 (-7.06,-1.4) | 0.003 |
| BMI | -0.39 (-0.44,-0.34) | < 0.001 |
| Waist | -0.14 (-0.16,-0.11) | < 0.001 |
| SBP | 0 (-0.03,0.03) | 0.946 |
| DBP | -0.11 (-0.14,-0.08) | < 0.001 |
| Ghb | -0.97 (-1.59,-0.34) | 0.003 |
| Cpeptide | -5.12 (-6.11,-4.14) | < 0.001 |
| Insulin | -0.16 (-0.2,-0.13) | < 0.001 |
| CRP | -2.57 (-3.18,-1.95) | < 0.001 |
| ALB | 0 (0,0) | 0.785 |
| CR | 0.01 (0.01,0.01) | < 0.001 |
| Wbc | -0.57 (-0.75,-0.39) | < 0.001 |
| Rbc | 4.85 (4.2,5.51) | < 0.001 |
| Hgb | 1.94 (1.73,2.16) | < 0.001 |
| TyG index | -1.81 (-2.36,-1.27) | < 0.001 |
